# An ‘arsenal for the supply of ammunition for the defence of vaccination’: the Jenner Society and anti-anti-vaccinationism in England, 1896–1906

**DOI:** 10.1017/mdh.2024.28

**Published:** 2025-01

**Authors:** Matthew L. Newsom Kerr

**Affiliations:** Santa Clara University, Santa Clara, CA, USA

**Keywords:** Anti-Vaccinationism, Jenner Society, Vaccine Advocacy, Medical Misinformation, Medical Expertise, Francis T. Bond

## Abstract

Although historians have given close attention to the anti-vaccination movement that gripped late-Victorian England, relatively little scholarship explores how doctors and health officials responded or asks what strategies and assumptions structured how they might oppose the vaccine opponents. This article traces the advent and actions of the Jenner Society, a smallpox vaccination advocacy group founded in 1896 by Dr. Francis Bond. His goal was to publicly confront the leading anti-vaccinationists and to effectively conduct an *anti-anti-vaccination* campaign. The Jenner Society appeared amidst disputes over how and even whether vaccination should be publicly debated – disputes shaped both by long-standing attitudes toward professional propriety and also by indecision about what sorts of political advocacy were suitable for medical practitioners. Vaccination was shifting toward a more voluntary administration, and the Jenner Society represents how civil society, the popular press, and the modern tools of persuasion were becoming increasingly central to public health governance. The Jenner Society encapsulated the profession’s disdainful attitude toward populist medical dissent, and this essay argues that the tone and deportment of anti-anti-vaccinationism had the effect of encouraging doctors to overlook and neglect other, probably more significant, sources of vaccine skepticism. Preoccupied with rebutting and attacking vaccination’s enemies, public “controversialists” like Bond waged the first true large-scale pro-vaccination propaganda campaign, but they ultimately were unable to address the underlying dynamics of vaccine evasion. This history holds important lessons today for those interested in constructing more effective ways to effectively counteract medical misinformation and anti-vaccinationist beliefs.

## Introduction

Dr Francis T. Bond’s antipathy toward anti-vaccinationists likely dates from a disappointing public debate in 1884. As the Medical Officer of Health for the Gloucestershire sanitary district in the west of England, Bond saw it as his duty to refute the alleged harms of smallpox vaccination and defend the law which made it compulsory for all infants. An opposition campaign had begun a few years earlier by the influential proprietor and editor of the *Gloucester Citizen*, Samuel Bland, who was also the president of the local anti-vaccination society and Bond’s opponent in this particular debate. The sleepy cathedral city was emerging as a hotspot of non-vaccination and, despite later suffering through a devastating smallpox epidemic, would remain an important bastion of English vaccine scepticism and a point of pride for the National Anti-Vaccination Society. The 1884 debate underscored some of the challenges which doctors and surgeons faced when speaking out on vaccination and refuting medical misinformation. It featured a raucous crowd clearly predisposed to jeer Bond, who seemed out of his depth in that environment and struggled to parse the controversial aspects of the vaccination law. He pleaded that such a gathering was not conducive to gaining a reasonable understanding of the issue. Bland meanwhile easily gained the upper hand, his disdainful and droll performance eliciting roars of approval, according to his own paper’s account of the event.^1^

Although Bond never again ascended a stage to defend vaccination, he did go on in 1896 to found the Jenner Society – a group devoted to carrying on the dispute through popular literature and newspaper letter-writing.^2^ As its honorary secretary, Bond emerged as the most prominent vaccination ‘controversialist’ of the day, obstinately waging war on anti-vaccinationists in the English press. For too long, he explained in a letter to *The Standard*, it had not been thought necessary to respond to their ‘organized propaganda’, to ‘counteract this attack’, or even to explain to parents why their children should be vaccinated. ‘We have all been living in a fool’s paradise, solacing ourselves, apparently, with the ancient fallacy that truth is great and will prevail; but forgetting the teachings of all history that it never does so, except at the cost of energetic struggle to establish and maintain it’.^3^ The Jenner Society was conceived as a counterpart to the National Anti-Vaccination Society, devoted to studying its methods and establishing ‘a corresponding organization for [vaccination] defence’.^4^ This new campaign was straightaway perceived as a battle for the public mind, putting vaccination advocates on offence. *The Pall Mall Gazette* rejoiced that at last, ‘Propaganda was to be met by propaganda’.^5^ Bond wanted not only to promote vaccination but also to actively engage in the types of acrimonious confrontations that doctors had long avoided. The style of advocacy that personified the Jenner Society can best be described as *anti-anti-vaccinationism.* ^6^

Historians have paid a great deal of attention to the robust anti-vaccination movement of late Victorian England, working out its main grievances, constituencies and ideological factions, tactics of organised resistance, and political cultures of the body.^7^ At the same time, rather little has been asked about how doctors and health officials responded and what strategies and assumptions helped to assemble but also constrain vaccine advocacy. To be sure, the Jenner Society’s brand of anti-anti-vaccinationism was just one, short-lived mode of combative engagement, a mirror image of what doctors perceived to be the National Anti-Vaccination League’s tactics of public interpolation. By the 1890s compulsory smallpox vaccination was perhaps the most contentious medical issue in the English-speaking world. Although they tended to avoid public confrontations on the issue, ordinary practitioners increasingly were forced to contend with questions concerning when and under what circumstances vaccine science should be debated, whether to undertake acrimonious disagreement with laypersons, and how professional authority might be leveraged to influence popular opinion. The Jenner Society arrived on the scene just as the law on smallpox vaccination was shifting from a compulsory regime to a more voluntary one. Active for just under a decade, its work also exemplifies how the medical profession struggled to adjust to these new political realities and navigate the climate of controversy around vaccination that already pervaded public discourse.

The difficulties experienced by the Jenner Society bear some resemblance to more recent concerns about the gap between professional consensus and popular apprehensions. In 2019 the World Health Organization declared vaccine hesitancy one of the top ten threats to human health – a warning that was tragically prescient in some countries during the COVID-19 pandemic.^8^ Reluctance and resistance to vaccinations undoubtedly arise from complex and multiple factors. Nevertheless, many discussions tend to attribute vaccine hesitancy almost entirely to the activities of certain personalities or so-called misinformation networks, seemingly stocked with professional sceptics, conspiracy theorists, fringe ideologues, and bad-actor opportunists.^9^ From this perspective, a solution has been to more effectively police public discourse, moderate social media content, and counter-program or de-platform the so-called ‘super-spreaders’ of medical misinformation.^10^ Another theme among critics of vaccine hesitancy emphasizes broad cultural shifts and societal sensibilities, with some commentators warning about the ‘death of expertise’ and a ‘war on science’.^11^ In a somewhat different vein, others have drawn upon the psychology of cognitive bias and negative polarization to explain vaccine wariness, with the hope of improving science communication and developing programs of trust-building that employ shared values.^12^ This article does not focus on vaccine hesitancy or opposition *per se* but rather deals with the medical profession’s struggle at the turn of the last century to comprehend and react to the phenomenon. A handful of doctors decided to wage war on the National Anti-Vaccination League. How and why these anti-anti-vaccinationists fell short of their goals can yield important lessons for the ways that we understand the complicated tensions between vaccine advocacy, medical authority, and public engagement.

## ‘Inconsistent with self-respect’

So much attention has been directed at the rise and impact of the Victorian anti-vaccination movement that scholars have tended to neglect the doctors’ response. Doubts and objections to vaccination were raised at the beginning of the century, but an organised and popular resistance movement did not take hold in England until the Vaccination Act of 1853 introduced the infant vaccination requirement. An 1867 amendment provided for stricter enforcement, including legal prosecution and fines for non-compliant parents. Anti-vaccinationists (as they now called themselves) included a variety of medical dissenters, such as John Gibbs, a hydropath whose inflammatory pamphlets claimed that vaccination, besides not preventing smallpox, caused bodily degeneration, conveyed unwanted diseases, and resulted in skin ulcers and death.^13^ These fears suddenly gained prominence after lying dormant for decades, and officials like the Medical Officer of the Privy Council, John Simon, were not sure what to make of them. His 1857 treatise laying out the rationale for state-enforced vaccination described Gibbs and his ilk as ‘a kind of literary fossil’, reminding one of ‘something so utterly unpractical, ante-diluvian, and extinct, that the last act I would think of committing against them, would be to argue’.^14^ And so he did not.

Although mainly perplexed by vaccination opponents, physicians and surgeons like Simon were also perturbed by their crass impertinence, which challenged newly won legal recognition of medical qualifications.^15^ Furthermore, codes of professional propriety limited doctors and surgeons to respectable channels of influence and ruled out participating in antagonistic disputes with obscure critics. Dr Edward C. Seaton, the top vaccination expert for the Medical Department of the Local Government Board, worried that refuting the anti-vaccinationists’ ‘worthless diatribes’ would lower the dignity of the profession.^16^ One doctor’s pamphlet stated that condescending to disprove these ‘utterly irrational or intellectually dishonest’ arguments would be ‘inconsistent with [his] self-respect’.^17^ Most therefore felt no special compunction to explain or justify vaccination to the public. The professor of pathology at the Army Medical School found it ‘almost an insult to human understanding to be obliged to collect statistics to *prove* that vaccination’ protected against smallpox.^18^ Expertise and standing, in other words, should translate directly into authority and confidence. In fact, Simon suggested that it was not the doctor’s position to wrestle with distrust. Rather, it was the business of the law to conquer the people’s indolence, credulity and apathy.^19^ The professional consensus seems to have been to adopt a policy of firm patience, depending upon the vaccination mandate to mop up the negligent and recalcitrant. A similar attitude was held regarding the anti-vaccination movement. One Scottish doctor wrote that this ‘crotchety’ crusade ‘may be safely left to itself, its tendency to early death from collapse being manifest’.^20^

By the 1880s, however, anti-vaccination had taken off as a campaign encompassing many different critiques and having the ability to influence a wide set of persons who would not necessarily consider themselves part of a movement. As Nadja Durbach has shown, it was the prosecution of parents that created a lightning rod for sympathy, fuelled membership in local support leagues, and evoked indignation at public meetings and demonstrations.^21^ In Leicester in 1885 an assembly of at least 80,000 participants witnessed Jenner hanged in effigy. Despite evidence of substantial genuine discontent toward the intrusiveness and potential harms of vaccination, English physicians generally chose to attribute rising non-vaccination rates to the ‘pernicious propaganda’ circulated by organised groups.^22^ This stream of pamphlets, tracts, leaflets, and magazines was often denounced as another unintended evil of mass literacy, akin to the risqué novels, obscenity, and other moral poisons ushered in by the cheap press. Echoing well-worn stereotypes about plebian impressionability, Dr Seaton complained that anti-vaccinationist literature was styled so as to lead working-class readers to accept its assertions ‘without reflecting on the matter at all’.^23^ There certainly was a lot of it. William Gladstone remarked how the tables of members of parliament (MPs) ‘groan under [its] weight’,^24^ and the Duke of Northumberland quipped that he had received ‘a sufficient supply of [anti-vaccination] pamphlets to start a successful business in waste-paper if he so desired’.^25^

The government also did not see much of a role for itself in the growing controversy. Local authorities could expend public funds for literature promoting vaccination, but few apparently did, and often only when an epidemic threatened. An 1869 editorial in *The Saturday Review* grumbled that the surge of anti-vaccination texts (‘couched as they usually are in the most violent and unintelligible language [and] largely circulated among the poor’) went almost completely unanswered. It pleaded for the Privy Council to create an official tract to refute these misrepresentations, so that at least ‘the agitators would no longer have things their own way’.^26^ Not until several years later did the Local Government Board give its sanction to a broadsheet issued by the National Health Society. This however was immediately followed by one published by the London Society for the Abolition of Compulsory Vaccination purporting to refute it point by point.^27^ The Health Society’s pamphlet was later attacked in Parliament by an anti-vaccination-allied MP for containing ‘monstrous falsehoods’, prompting a minister to distance the Government from the publication and signal that it did not want to be considered trying to influence public opinion on the issue.^28^

Victorian-era anti-vaccination literature was nimble, plentiful, and pitched to the people. It also had no real equivalent, or even anything approaching an equivalent, for vaccination supporters. Popular texts written by doctors defending vaccination did appear, but these were infrequent, unsystematic, and generally unremarkable. They also typically missed the mark in appealing to a popular readership. An example is *The Truth of Vaccination* (1880) by Ernest Hart, the editor of the *British Medical Journal* and leading sanitary reformer. Hart stated upfront that he had no intention of imitating the ‘inflammatory postcards, grotesquely drawn envelopes, and other means of disseminating [anti-vaccination] views’ which ‘circulated industriously and persistently amongst the classes … least able to judge for themselves’.^29^ His book was intended for legislators and ‘public men’ who lacked a handy compendium of medical teachings on vaccination efficacy and safety; however, it veered into obscure points of pathology in a way that suggests that Hart did not have in mind a clear idea of his audience – a common weakness in publications of this sort.

Anti-vaccinationist beliefs and non-vaccination practices both grew slowly and steadily. In 1873 less than 5% of infants in England and Wales went unvaccinated by the required age of three months; by the end of the century, this amounted to roughly a third of children. Default rates were even higher in certain communities where vaccine defiance was able to sink deep roots into local cultures of dissent – notably Midlands towns such as Leeds, Keighley, Oldham, Halifax, and Leicester, where local campaigners could count on the assistance of the National Anti-Vaccination League, with its cadre of paid agents, lecturers, and letter writers.^30^ Opposition (or at least uneasiness) toward the Vaccination Act grew as a minority faction within the Liberal parliamentary party, but its enforcement was a local matter, and thus activists also pushed to capture key parish and municipal posts. An anti-vaccination hotspot typically started with a local newspaper editor denouncing the prosecution of refractory parents and helping to disseminate vaccine scepticism. Meanwhile, agitators would demand electoral pledges opposing prosecutions, sometimes ‘button-holing’ both political parties on the issue and causing the Vaccination Act to become a virtual dead-letter. Activists at times credited the inaction of local doctors for their success. One Leicester anti-vaccinationist was astounded by ‘the almost entire absence of any serious attempt to convert the opponents of vaccination either by letters in the press or by lectures and public discussion’.^31^

This general pattern played out in Gloucester for a decade leading up to its devastating smallpox epidemic. In 1887 the Board of Guardians voted to cease all prosecutions of parents for non-vaccination, and within a decade nearly 85% of children in Gloucester went unvaccinated. The rapid rise of smallpox in early 1896 led rattled city officials to issue a tepid statement recommending the vaccination of unprotected children; it was enough to enrage local anti-vaccinationists. A large indignation meeting was treated to a two-hour speech by Dr Walter Hadwen, an agent of the National Anti-Vaccination League famed for his gripping oratory. Although a qualified practitioner, Hadwen adopted unorthodox treatments and was vilified as an apostate by the medical establishment. He delivered all the typical elements of the anti-vaccination creed: questioning its ability to prevent smallpox, highlighting supposed cases of injury and death from ‘blood-poisoning’, upholding the ability of sanitation to preclude all epidemics, and denouncing the Vaccination Acts as an intolerable interference with liberty.^32^ The League circulated leaflets and posters advising Gloucestrians to shun the smallpox isolation hospital (that ‘overcrowded pest house’) and treat the sick in their own homes.^33^

Smallpox in Gloucester quickly grew out of hand. By early April, with most aspects of public life grinding to a halt and the city’s situation garnering nationwide anxiety, the parish Guardians voted to resume enforcement of the Vaccination Law. During the height of the panic, a special committee headed by Dr Francis Bond went door-to-door and performed nearly 35,000 vaccinations and revaccinations in just two months (out of a population of 42,000). It suddenly transformed Gloucester from one of the worst to the best-vaccinated town in England. There was probably no record anywhere, Bond wrote, ‘of so rapid, so extensive, and so momentous a conversion on any matter not directly connected with a question of religious belief’.^34^ He and others credited the mass vaccination campaign with bringing the epidemic to a close. And indeed, for a while, it seemed as though the local vaccine resistance had crumbled to pieces. Yet even after 434 deaths and at least 1,981 infections, anti-vaccinationism continued to exert a powerful influence over Gloucester’s politics. At the next election, leading anti-vaccination candidates swept all contested seats on the City Council and Board of Guardians. The following year Dr Hadwen was elected at the head of the poll for the city School Board and enforcement of infant vaccination was again repealed. Non-vaccination rates in Gloucester infants steadily rose: 45% in 1909, 60% in 1912, and over 80% throughout the 1920s.^35^

## To ‘disarm the objectors’

The searing experience of the Gloucester epidemic gave birth to the Jenner Society and wholly shaped its style of vaccine advocacy. Bond had previously reached out to several local dignitaries for the centenary celebration of Edward Jenner’s initial experiments with vaccination, but the smallpox outbreak prompted a major change in focus. The Jenner Society’s prospectus, issued in April 1896 at the height of the crisis, stated that the primary objective would be counteracting ‘the mischievous efforts so persistently made to discredit the name and work’ of Jenner and ‘bring[ing] home again to the mind of the nation, in a time of growing forgetfulness of his great discovery, the immense benefit he conferred by it upon mankind’. This involved two main activities: firstly, measures to ‘collect, diffuse, and popularize’ knowledge about the history of smallpox and the effectiveness of vaccination. Secondly, efforts to ‘systematically reply to and expose the misstatements and fallacies’ made by anti-vaccinationists, ‘to the neglect of which hitherto is mainly attributable the hold which this mischievous agitation has obtained on the public mind’.^36^

From its outset, the Jenner Society was envisioned to serve as a counterweight to the National Anti-Vaccination League. The Executive Committee’s first report complained that public discourse for years had been ‘systematically poisoned’ by persons who ‘could not or would not recognize the mischief they were doing’. Rescuing vaccination from oblivion thus required providing an effective ‘antidote’.^37^ Again, the intent included far more than mere vaccine promotion. Bond’s colleague in London, Dr Albert Cope, argued that the last hope for smallpox vaccination in England resided in ‘systematic, organized education on the subject’. This meant ‘plain and unmistakable presentation of the truth about vaccination’, and it necessarily involved ‘a persistent warfare against misrepresentations – if not worse – of the anti-vaccinationists’.^38^

The Jenner Society organisers clearly intended to rouse the medical profession for the defence of vaccination but were wary of advertising it as a doctors’ association. Bond had started by recruiting ‘all the leading men of Gloucestershire’ and soliciting the support of national scientific celebrities like Lord Lister, internationally known philanthropists such as Andrew Carnegie, and the novelist H. Rider Haggard. Significantly, though, top honorary posts were reserved for the Presidents of the British Medical Association and the General Medical Council, with substantial donations of support coming from the BMA, the Society of Medical Officers of Health, and the Royal College of Physicians of Edinburgh. Thus, while pitching itself as a broad-based civil society group, the Jenner Society stepped forward as an instrument for rectifying the aloofness of the nation’s practitioners who, according to one highly critical editorial on the vaccination issue, had shown ‘gravely mistaken regard for the dictates of etiquette or *amour propre*, in preference to those of civic duty’.^39^

The issue reached a critical juncture in 1898 with the passage of a new Vaccination Act for England and Wales. The act produced considerable consternation amongst rank-and-file doctors because it allowed parents for the first time to claim a ‘conscientious objector’ exemption to the infant vaccine mandate. But the Jenner Society, to the surprise of many, actually played a key role in bringing it about. The final report of a Royal Commission on Vaccination had recently declared smallpox vaccination overwhelmingly safe and effective, yet advised that better results might be had if enforcement of the mandate were made more conciliatory. In speeches and pamphlets, Bond put considerable effort into stressing the point: the principle of a vaccine mandate was still sound; the problem was that its uncompromising application had become simply ‘impracticable’.^40^ His main concern was that prosecution and punishment of refractory parents only lent fuel to vaccine opponents. This logic made its way into the final bill and, as enacted, the key and controversial portion of the 1898 Act upended decades of infant vaccination enforcement. Penalties for failing to vaccinate a child could be dismissed if its guardian appeared before a magistrate and declared a ‘conscientious disapproval’ of vaccination.^41^

*The Times* commended Bond as a physician who possessed ‘an Englishman’s love of liberty, even when it is misguided’.^42^ It would be more accurate to view Bond’s backing for the ‘conscience clause’ as a continuation of his crusade against anti-vaccinationists. Indeed, Jenner Society memoranda unapologetically describe it as a means of outmanoeuvring the National Anti-Vaccination League, essentially depriving this small band of noisy extremists of the platform given to them by prosecutions and thereby insulating the majority of parents from their cries of martyrdom.^43^ Although he framed it as a generous concession to honest objectors, Bond left no doubt he believed that the result would be to make vaccination less avoidable for everyone else. Being obliged to claim an exemption would screen out the merely ‘indifferent and indolent’ parents, who could still be prosecuted.^44^ Admittedly, the approach presented a delicate balancing act: how to ‘disarm the objectors’ without opening the door to wholesale evasion of vaccination.^45^ This was made more difficult by the fact that the 1898 Act failed to include measures that Bond considered essential for its success, such as fixity of tenure for public vaccinators, a national standard of efficient vaccination, school-attendance conditions, and requirements for systematic re-vaccination at age twelve.^46^ Furthermore, the law did not constitute an unambiguous Government endorsement of vaccination, nor did it make an official commitment to ‘protect the public against the misconceptions’ spread by anti-vaccinationists.^47^ Top ministers believed they had brokered a compromise and were unwilling to wade further into a medical question that aroused bitter public controversy. The result was that it became even more difficult to insist on the absolute necessity of vaccination while simultaneously creating ways to evade it.

Thus, far from settling the issue, the 1898 Act created a highly uncertain future for vaccination, with all sides nervously awaiting the outcome. It also thrust an even heavier burden for defending vaccination onto civil society groups. Speaking for the Jenner Society, Dr Cope urged practitioners to acknowledge that vaccination could no longer be simply commanded. As he explained, ‘we must now recognize the change in political conditions, and the awakening of democratic instincts, and to succeed must “educate the democracy”’. Cope however paired this appeal with a dire warning, predicting that vaccination did not stand a chance unless supporters united to wage a last-ditch ‘crusade’ against their adversaries.^48^

## ‘Take up the cudgels against the antivaccinists’

Bond almost immediately found that the bulk of his duties as the Jenner Society’s honorary secretary consisted of personal correspondence to newspapers disputing the statements of anti-vaccinationists. It was a form of engagement he had honed in the Gloucestershire and Bristol press as a sharp-witted commentator on local sanitary matters and Home Rule debates. In other respects, he was an unlikely public figure. Bond published occasionally on matters pertaining to pathology, chemistry, and sanitation. From 1873 he served as the medical officer for the rural sanitary district of Gloucestershire (a position he held until his death in 1911). He also established a dairy and cheese manufactory in Gloucester, held several patents, and avidly promoted the health benefits of skipping rope.^49^ Some of Bond’s own supporters questioned the wisdom of directing the crusade to save vaccination from ‘such an out-of-the-way part of the world’.^50^ He nonetheless proved an extremely prolific contributor to the vaccination debate, regularly publishing more than one hundred letters to the editor per year, many of considerable length and involving multiple exchanges. Bond reported that in 1898 over 250 letters and articles by Jenner Society members had appeared in newspapers across the kingdom.^51^

By this time the major London and national newspapers had generally closed their correspondents’ columns to anti-vaccinationists, but this was not the case with many provincial ones. Bond complained that their editors foolishly treated vaccination as if it were still a fairly divided controversy and asked whether they should accept the responsibility of allowing discourse that was ‘in the highest degree mischievous’. It was in the public interest, he urged, that ‘the line should be drawn somewhere’, especially with letters simply rehearsing frequently debunked arguments.^52^ Vaccine advocates for years had urged editors to stop giving space to anti-vaccinationist opinions because, in the words of one doctor, it unwittingly helped to ‘deceive your defenceless readers’.^53^ Several newspapers did indeed decide to shut their columns to discussion of the controversy, sometimes when an epidemic seemed imminent. This was the case with *The Cambrian* in Swansea, which suppressed duelling correspondences about vaccination in 1902 after cases of smallpox occurred in town. ‘[W]ith the enemy at the gates it would be suicidal to publicly discuss the weaknesses in the defences’, the editor declared.^54^ Some libraries and institutions, such as Toynbee Hall and the People’s Palace, refused to hold or lend anti-vaccinationist books and journals.^55^

In the meantime, Bond kept up a very considerable correspondence addressing the same disputes over and over again, his personal goal being to allow no anti-vaccinationist misstatement to go unanswered in the public press. The scale was massive, the Anti-Vaccination League having for years made newspaper correspondence a ‘stock method’ of propagandism. At one time there were reportedly over one hundred members in its Literary Union, all of whom pledged to place at least one letter a month to a newspaper questioning vaccination.^56^ A *British Medical Journal* editorial remarked that few had noticed how this ‘epistolary claque’ had manufactured an ‘underground current of misrepresentation slowly but surely permeating the soil of public opinion’. The solution, according to a Jenner Society report, was to systematically contradict it. There was ‘no better way of educating the public’ than showing the reading public that these reckless assertions could be ably refuted.^57^ Bond appealed to doctors to send him newspaper cuttings that needed answering and he employed a clipping service to reconnoitre the public press.^58^

Bond’s letters usually focused on disputing historical, physiological, statistical, and epidemiological facts, while he also gave his opinions on the rationale for government mandates, the ethics of parental consent, and the weight of professional consensus. Depending on the publication and editorial discretion, some of these exchanges were little more than vituperative banter and others were profoundly petty. And to be sure, Bond’s acerbic writing style clearly irritated his adversaries. A pamphlet issued by the Gloucester anti-vaccinationists mocked his ‘pedantic verbosity’.^59^ Those he squared off with in newspaper columns sometimes protested that Bond’s ‘dishonest methods of controversy’ were ‘aggressive’ and ‘very bitter’.^60^ One complained of a ‘venomous personal attack’ and ‘abusive bluff’, while another accused him of ‘undisguised contempt’ toward anti-vaccinationists.^61^

Bond’s ubiquity in the major and minor English newspapers made him arguably the most prominent vaccination advocate of the day. *The Times* portrayed Bond as an ‘untiring’ thorn in the side of anti-vaccinationists.^62^ Many years later others would recall his ‘fearless disposition’ as an ‘inexorable adversary of the anti-vaccinationists’.^63^ He was clearly also the driving force behind the Jenner Society, which fuelled criticisms that the anti-anti-vaccination campaign was the product of his singular personality. Hadwen quipped that ‘Dr. Bond is the “Jenner Society” and the “Jenner Society” is Dr. Bond’. The group held no public meetings and could point to no popular base of support – it was a mere ‘paper society’.^64^ There was some truth to these assertions. The Jenner Society, although it published an annual list of supporters and developed a network of distributors for its literature, did not establish local branches or have any perceivable existence apart from the written record. Yet Hadwen’s fellow activists evidently still viewed the Jenner Society as a genuine threat. In 1899 the National Anti-Vaccination League launched an appeal for £5,000 to a special fund intended to check its actions.^65^ The Jenner Society’s annual budget, meanwhile, only once exceeded £200.

Bond did not seem to mind the notoriety of being a public controversialist. But he also offered assistance to any doctors similarly ‘prepared to take up the cudgels against the antivaccinists’.^66^ The *British Medical Journal* applauded this suggestion, contending there were times when it became sensible ‘to answer the fool according to his folly’.^67^ In the main, though, Bond’s challenge ran up against a considerable amount of wariness in the profession over the means of publicity and the costs of conflict. *The Lancet*, for example, did not think it *‘*either desirable or seemly’ for doctors to take prominent roles in ‘propagandism’.^68^ It also observed the hostility and ‘violent language’ that often greeted those who challenged the anti-vaccinationists in print or on the debate stage. There was, the *Lancet* editorial writer warned, ‘hardly any subject in which personal vilification has more frequently sufficed to take the place of argument’.^69^

The Jenner Society was nothing if not alive to the difficult task of managing an emotionally explosive public controversy, and Bond encouraged rebutting anti-vaccinationist statements wherever possible in print. Participating in public debates with anti-vaccinationists was deemed risky and he personally advised against it. ‘We have had some experience of the tactics of anti-vaccinators at such meetings’, he once tersely replied.^70^ There was something about a crowd of people, Bond went on to explain, that favoured the anti-vaccination agitator, who ‘Like the astute stage manager … marshals his buckram army of garbled facts and egregious fancies before the dazzled eyes of the bewildered spectator until they become invested with an importance out of all proportion to their real strength’.^71^ Bond’s description reveals how he assumed anti-vaccinationism to be synonymous with theatrical tricks and illusions. By extension, medical debate and public debate were fundamentally incompatible. Many vaccination advocates, citing personal experience, agreed. The medical officer for Bradford proposed as a general rule to never publicly confront the anti-vaccination orator, since one could never beat the ‘stumper’ and his stream of outrageous misstatements.^72^ Their events could also attract ill-tempered counter-demonstrations, as in 1909 when students from the local medical school attempted to break up an anti-vaccinationist meeting in Bristol, heckling and making havoc during a speech by Dr Hadwen.^73^

This did not rule out public lectures, though, as seen in the series of appearances around England in 1899 by Dr Elizabeth Garrett Anderson, the first woman to qualify as a doctor in Britain, who toward the end of her professional career lent considerable clout and celebrity to the vaccination cause.^74^ Garrett Anderson went on to head up another organization devoted to Parliamentary lobbying, the short-lived Imperial Vaccination League. It too maintained a strict policy against public debating. In declining one challenge, her secretary wrote that ‘vaccination, as a scientific question, depending on evidence open to all, is not a fit subject for debate with pronounced opponents on a public platform. The hearers are apt to be influenced more by the eloquence and fervour of the speakers than by the weight of the evidence’.^75^

Anti-vaccinationists meanwhile were wont to frame this as an evenly-sided dispute, and touted their eagerness for stage debate as proof that they were the only ones in favour of ‘free and open inquiry’.^76^ They excelled in throwing out challenges, even wildly unrealistic ones. For instance, in 1887 Mr Alfred Milnes dared the entire British Medical Association, twenty-six thousand strong, to ‘produce a champion for public debate’.^77^ A prominent East End anti-vaccinationist offered the Bishop of Stepney to debate whether vaccination was a sin.^78^ Bond and his colleagues perceived these to be little more than empty taunts and part of the anti-vaccinationists’ propaganda strategy. Nonetheless, doctors’ reluctance to publicly debate became a standard talking point in anti-vaccinationist literature. Bond’s nemesis in Gloucester, Dr Hadwen, for years ridiculed him as terrified of attending any public meetings in the city and argued that this alone was strong evidence of the weakness of the Jenner Society’s case.^79^ There was undoubtedly an intimate connection between public speech and public life in the later Victorian period. The era’s culture of political debate exalted platform oratory as the embodiment of modern democracy and the rational discourse that constituted the public sphere.^80^ This platform culture was undergoing significant changes toward the end of the century, marked by the appearance of new social actors and speakers, such as women and working men, who challenged the priority usually given to ‘public gentlemen’. These were, in essence, contests over who deserved a platform. Many doctors meanwhile questioned whether accommodating a controversial speaker contributed to the public good and if public confrontations were fundamentally ill-suited to addressing medical misinformation.

Still, whether on page or stage, the Jenner Society’s crusade sought to emulate the stratagems of battle. Repulsing the anti-vaccinationists required courage, careful planning and tact, Bond wrote: ‘It is a form of warfare which needs some little training to ensure success; for the enemy is crafty, is familiar with the use of his weapons, and too often does not mind striking a foul blow when a fair one fails’.^81^ He also worried that doctors and surgeons too often simply lacked an appreciation of their opponents’ polemic devices. In other words, they did not understand the fight which lay in store for them. Anti-vaccination agitators had learned to organise their attacks so as to disguise their lack of knowledge; they were masters of repeating their ‘patter’ so as to distract from their weakest points. Bond held that the same moral had been learned in the South Africa War, where the Boer’s ‘slimness’ was more than a match for the Britisher’s ‘frontal attacks’, until the latter studied his opponents’ deceptive tactics.^82^ The art of waging war upon anti-vaccinationists, Bond advised, was to avoid ambush. He recommended that all doctors familiarize themselves with the ‘controversial aspects’ of the vaccination question. The Jenner Society published a series of notes meant to prepare the reader to rebut the anti-vaccinationist stock arguments whenever encountered. A library of anti-vaccination literature was also kept for loaning to Jenner Society members, the idea being that to effectively fortify vaccination one needed to ‘study carefully the arguments urged against vaccination. In this way, they can be easily met’.^83^

## ‘Evangelizing the heathen in the truth of vaccination’

At its founding in 1896, the Jenner Society entertained several high ambitions, such as producing a journal dedicated to promoting vaccination and funding a vaccine and bacteriological laboratory in Gloucester modelled on the Pasteur Institute.^84^ There was at one time an effort to hire a speaker and organiser who could travel the country giving lectures, holding meetings, and establishing branches of the Society. The financial state of the Jenner Society never allowed any of these schemes to come to fruition. But indeed, there also seems to have been only modest enthusiasm for them. Without a doubt, the bulk of the Jenner Society’s organizational energy was put toward establishing a clearing house of literature for mass consumption, especially of the sort ‘calculated to meet the commoner forms of misrepresentation’ made by vaccine opponents.^85^ As this would suggest, the goal was not simply to distribute pro-vaccine messages and information; rather, Bond and his colleagues wanted resources that would unambiguously counteract anti-vaccinationist literature, which had for too long gone ‘uncombatted’.^86^ Tracts, pamphlets, leaflets, posters, circulars, and handbills constituted the war materiel of print culture battles, and this counter-crusade needed retaliatory weapons – ones as cheap, as plentiful, and as effective as their opponents’. Bond encouraged supporters to look upon the Jenner Society ‘as a sort of arsenal for the supply of ammunition for the defence of vaccination’.^87^

The Jenner Society published several original pamphlets and reprinted several articles and other pieces. Hundreds of thousands of shorter bills and circulars were disseminated for free or at cost. This had stemmed from Bond’s sense of disappointment that there existed practically no accessible literature which could expose the fallacies circulated by anti-vaccinationists. A few expensive treatises ‘of a more or less academic character’ had appeared from time to time, but ‘little to nothing that could be put into the hands of the general public’.^88^ The challenge was to translate the technical language of epidemiologists and health officials ‘into the language of the man in the street’.^89^ Bond wanted Jenner Society material to be the sort that doctors might set out in waiting rooms to be seen by patients and others.^90^ Some of it no doubt was ordered in bulk by persons like a clergyman’s wife who wrote to *The Lancet* expressing her need for ‘good serviceable literature’ on vaccination. Her house had been regularly ‘inundated’ with pamphlets from anti-vaccinationists, anti-vivisectionists, vegetarians and ‘kindred faddists’, and what she needed was ‘bright, telling tracts suitable for distribution, reading at mothers’ meetings, and for posting the clergy and others up in the incontestable [vaccination] facts and figures which are necessary to convince the obdurate’.^91^

The Jenner Society obliged with several different types of printed materials embodying a variety of messaging techniques. A common method was to summarise the positions of persons of stature and authority. Several leaflets consisted of reprints of editorials appearing in the medical press. One excerpted the forceful proclamations of Lord Herschell, former Lord Chancellor and President of the Royal Commission on Vaccination. In 1897 the Jenner Society arranged for a statement strongly supporting vaccination signed by over 1,100 Medical Officers in Great Britain, India, and the Colonies.^92^ A few handbills were little more than abridged summaries of essays that had appeared in professional and academic venues.^93^ A good portion of the Jenner Society’s literature consisted of timely material that sought to portray outbreaks such as at Gloucester as ‘object lessons’ justifying infant vaccination and adult revaccination.^94^ While having a penchant for prioritizing ‘the weight of authority’,^95^ as one leaflet bluntly put it, there was also an attempt to buoy the lay perspectives desired by the clergyman’s wife. A pamphlet authored by a Gloucester minister recounted his personal experiences during the epidemic.^96^ Another, titled ‘A Question of Conscience’, reprinted from the *Mother’s Union Journal*, featured an imagined conversation between a lady and her former servant, who has misgivings about vaccinating her baby but is ultimately reassured by her friendly persistence and the cogency of her arguments.^97^

**Figure 1.**
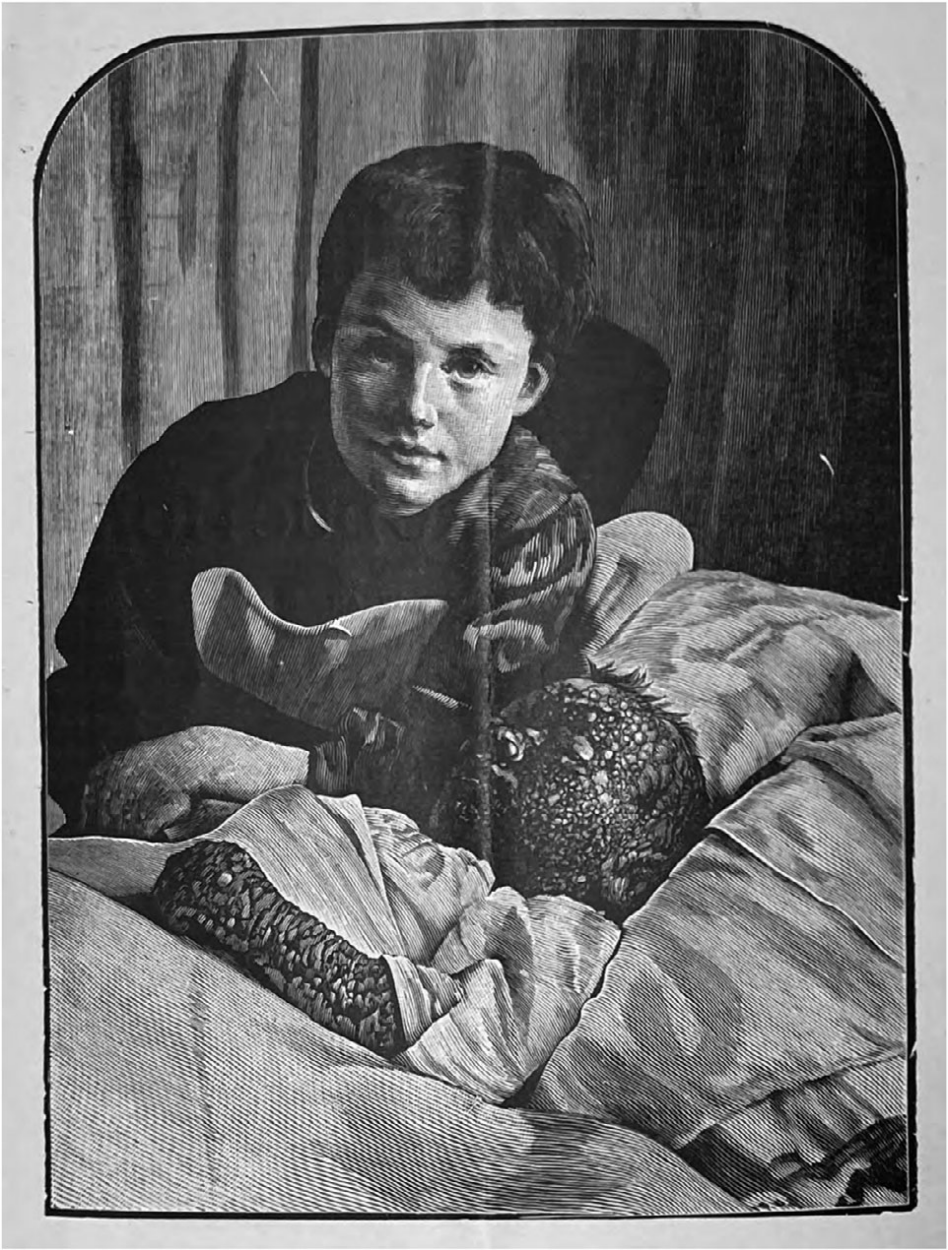
**Visualizing Anti-Vaccinationism.** GA/N27.20 *A Plea for the Children* (Gloucester: The Jenner Society, n.d.).

Reflecting the weight placed on Jenner’s legacy, these publications regularly sought to convey lessons about the history of disease. After a century of vaccination, another writer explained, the typical ‘man in the street’ very likely had never seen a case of smallpox. He does ‘not read much history, and when he does start to it he tackles the French Revolution or the Battle of Waterloo, so that his knowledge of what smallpox used to be remains practically nil’.^98^ One Jenner Society pamphlet from 1899, *A Plea for the Children*, described ‘small-pox as it was’ and ‘small-pox and it is’, arguing that the disease itself had not changed in its essence but its frequency had been suppressed by vaccination. It included photographs from pages of a register kept at Pudsey in Yorkshire in 1787 and 1792, showing smallpox’s terrible mortality in bygone times. With recent outbreaks like at Gloucester, the pamphlet goes on, persons today could ‘in some degree picture to ourselves what it used to be’. As if to literally aid that picturing, the pamphlet reproduces an arresting photograph from a smallpox hospital showing a lightly affected vaccinated girl leaning over the bed of her horribly affected unvaccinated brother^99^ (Figure 1). Smallpox should be seen as a disease of the past, the pamphlet suggests, and the unvaccinated body was a tragic and unnecessary relic of that history.

**Figure 2.**
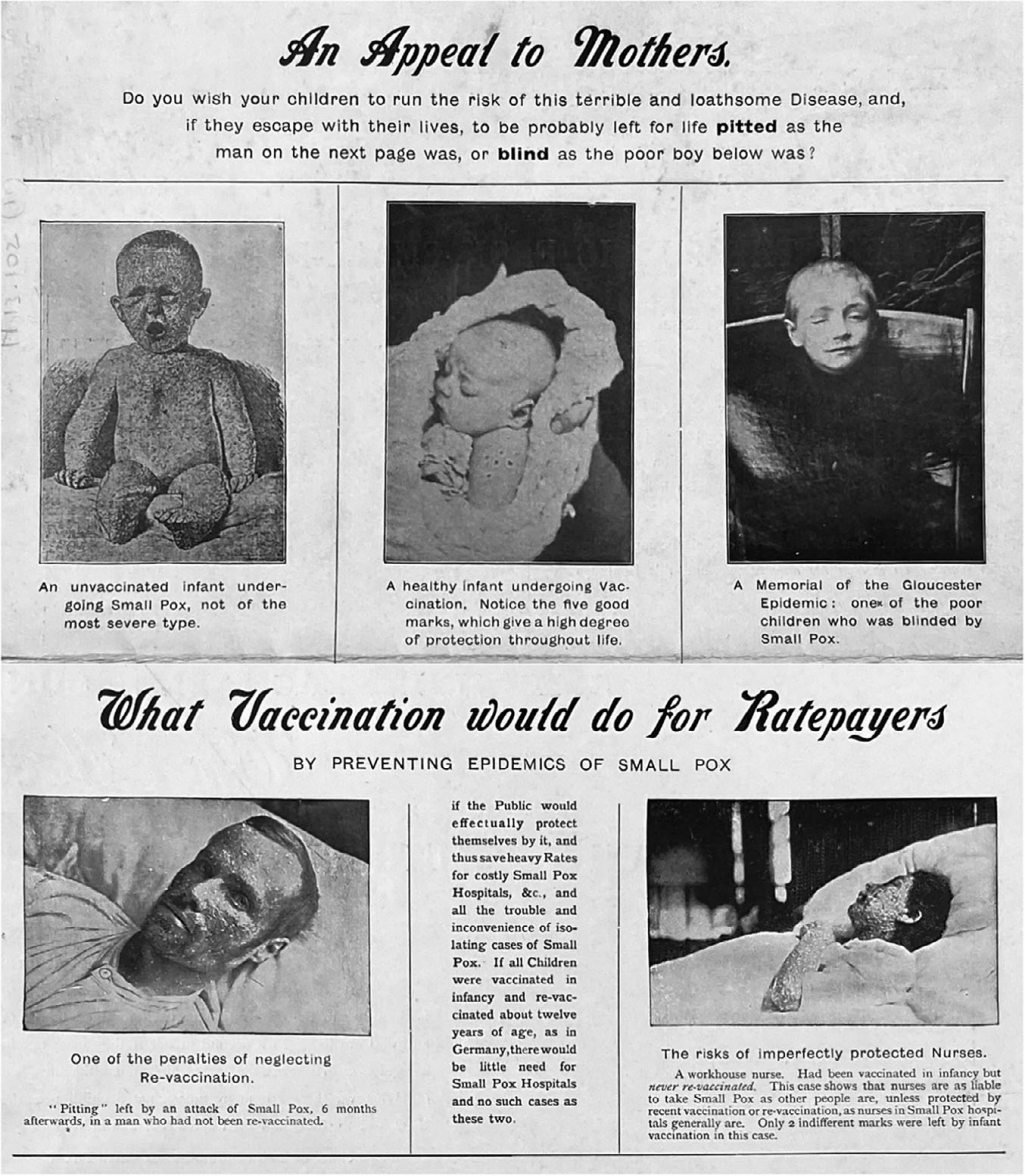
**The Penalties of Neglect**. GA/N13.102.11 Jenner Society, *Some Unimpeachable and Unanswerable Object Lessons in what Vaccination does to protect against Small-Pox in a series of Illustrations* (n.d.)

Jenner Society literature also made distinct attempts to imprint disease itself as the visual icon of anti-vaccinationism. This can be seen in a folded flyer described by the *British Medical Journal* as containing valuable ‘photographic object lessons’ in support of vaccination.^100^ The pictures show an unvaccinated sick infant, a boy blinded by smallpox, and a peaceful baby displaying characteristic vaccination marks. Other photographs depict a badly pock-marked man and a gravely ill nurse who had not been revaccinated (Figure 2). Another view shows a corner of the Gloucester cemetery, where 279 unvaccinated children were buried during the 1896 epidemic, the flyer explains, ‘through the criminal folly of their parents’. Mass-printed images, for technical and economic reasons, had previously been fairly marginal in the vaccination controversy. This was clearly changing at the turn of the century, another decisive factor being a shift in judging how best to agitate the public mind – and to some extent also gauging the public’s tolerance for painful images of disease. The Jenner Society offered to lend out a series of lantern slides exhibiting cases of smallpox for use in popular lectures. It was a device that the assistant Medical Officer for the County of Essex thoroughly appreciated, since in his experience vaccine opponents downplayed the disease as trivial and easily treatable. The pictures need not be ‘revolting in their character’, he explained, and they could not be more terrible than the disease itself, after all. Most importantly, it needed to be ‘brought home’ to the people how this dangerous and loathsome disease could easily be stamped out by vaccination. Illustrations might do more to convince sceptics than pages of written matter, he supposed, and ‘the anti-vaccinator will similarly be ‘stamped-out’ in time.^101^

This type of fear-based technique appeared alongside efforts to communicate complex arguments about social and personal risk. Much of the Jenner Society’s literature aimed to underscore the large body of statistical evidence in favour of vaccination, although the problem was still one of popular accessibility. Its early handbills and posters did a relatively poor job of presenting and summarizing these facts, and they likely required considerable study by the average reader to arrive at the desired conclusion. An article in the *BMJ* expressing the views of the Jenner Society (perhaps authored by Bond himself) admitted that the ordinary person was ‘often unable to appreciate, will scarcely even consider, averages, percentages, and other embodiments of numerical comparison. Like the “rule of three” in the old quatrain, they puzzle him, if they do not absolutely drive him mad’. Statistical figures often fell short precisely because of their impersonal, aggregate character, but most people could grasp the importance of a ‘concrete illustration’ about vaccination when it took the form of stories from individual experience.^102^

**Figure 3.**
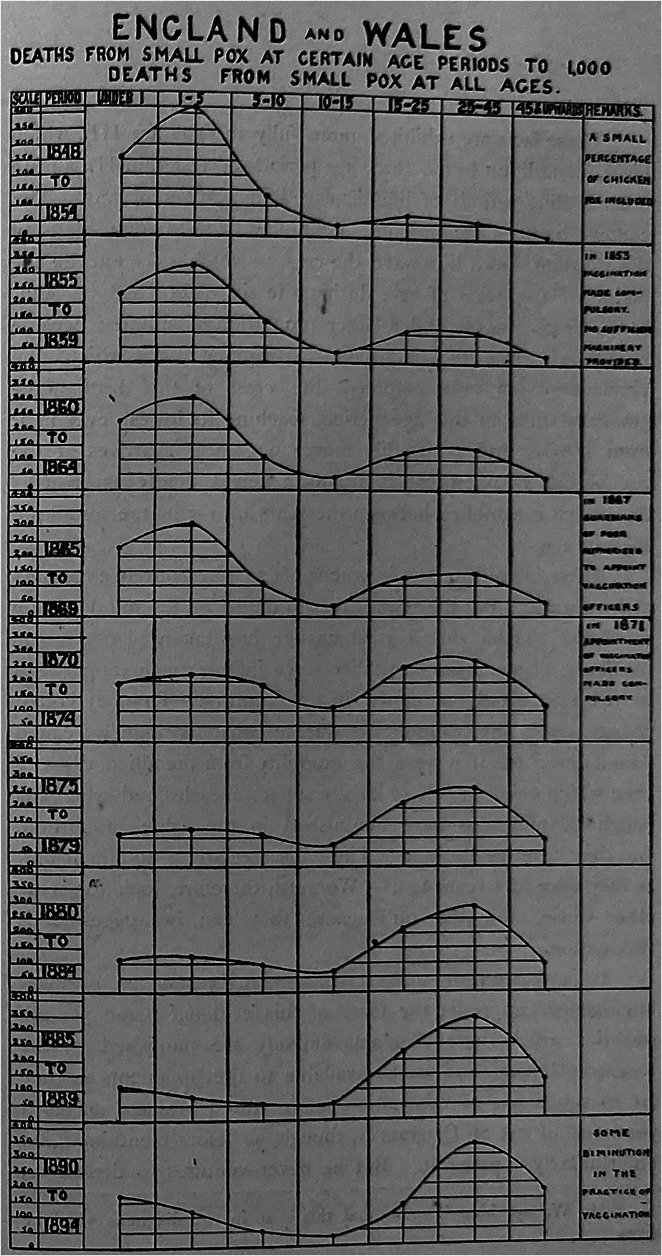
**Re-Presenting Statistics.** GA/N13.102.2 *The Effect of Vaccination in Infancy on the Mortality from Small-Pox in Childhood: A Critical Test* (Gloucester: The Jenner Society, n.d.)

Trust in numbers was not itself entirely built on representation, but their easy depiction could certainly help.^103^ ‘In these days of cinematograph displays and spectacular fever’, the London public vaccinator Albert Cope argued, ‘it becomes necessary for any cause to make its appeal directly to the eye, rather than to the mind’s eye’. To instruct the people, it was imperative to transform ‘shoals of statistics’ about the effectiveness of vaccination into presentations where ‘a glance of the eye may suffice to point to the moral’.^104^ Jenner Society literature gradually improved in its ability to illustrate concepts such as probability and disproportionality. This included reproducing graphics so as to visualize statistics in novel ways (which, incidentally, were completely absent from anti-vaccinationist publications), such as those appearing in abundance in T. D. Acland’s lecture series, ‘Vaccination and Common Sense’.^105^ An abstracted version of Acland’s lecture appeared as a Jenner Society pamphlet re-written into the kind of conversational language it claimed would allow the diagrams’ conclusions to be ‘so plain that ‘he who runs may read’ it’^106^ (Figure 3). An editorial in *The Spectator* argued that this was just what was needed. One must ‘*show*’ the people how vaccination is safe and effective because statistics alone ‘leave them just where they were’. The editorialist called for placards with these representations to be ‘posted on every wall’ and leaflets ‘handed in by every postman’, always taking care to associate the Government with them. ‘It is said that the Government stamp counts for something in the recommendation of doubtful medicines’.^107^

Bond however doubted that the government could carry out the sort of work he envisioned, this being necessarily of an aggressively polemical and propagandist nature. Officials rarely shared his enthusiasm for ‘evangelizing the heathen in the truth of vaccination’. Besides, the typical government office, with its ‘unavoidable formalities and restrictions’, he wrote, was not ‘best fitted to meet the not very scrupulous but very indefatigable activity of the [Anti-Vaccination] league’.^108^ Despite this, Bond did view the local state as a conduit for propagating the Jenner Society’s literature. In 1899 he received permission from Government authorities to distribute samples of Jenner Society pamphlets to numerous parish boards in the hope that they would purchase them in bulk. These went mainly to the west of England and Wales, but also to anti-vaccination hotspots like Leicester. The campaign met with mixed success. After Bond personally attended a meeting of the Chipping Sodbury Union, the Guardians decided to buy a selection of printed materials and large posters to distribute in parishes where the people were against vaccination.^109^ At the Barton Regis Union outside Bristol, the Union clerk stated that the pamphlet ‘illustrated with pictures with the children who had had smallpox, which were horrible’ would likely change many minds, but his Board decided not to purchase any, reasoning that there were few defaulters in the district.^110^ The Guardians in Leicester declined to even consider the offer, maintaining that it was not the duty of local officials to ‘become leaflet distributors to any organisation’.^111^ In Parliament an anti-vaccinationist-friendly MP severely criticized the Government for seeking to promote the Jenner Society’s ‘worthless and misleading publications’, and at least one Board of Guardians applied for permission to distribute publications of the National Anti-Vaccination League.^112^ Several commentators believed the Jenner Society had crossed a line. *The Councillor and Guardian* found that its methods were ‘rapidly bringing that small but fussy body into contempt’.^113^

## ‘A form of mental aberration’

Without any doubt, the Jenner Society’s members and supporters expended significantly more effort typecasting and deprecating anti-vaccinationists than seeking to understand the sources and causes of ordinary vaccine scepticism. Doctors and officials had long dismissed the leading ‘anti-vack’ agitators as ignorant laypersons, ill-mannered contrarians, or deceitful quacks – persons consumed by fallacies, falsehoods, fads, and follies; the ‘four F’s’, one medical critic asserted.^114^ By the 1890s, though, new psychological explanations were coming to the foreground, which strongly influenced how anti-anti-vaccinationists construed their own mission. For example, the Scottish physician and medical officer Dr John C. McVail remained puzzled by the obstinacy of the leading vaccination opponents. His *Vaccinated Vindicated* (1887) laid out a meticulous response to the writings of Alfred Russell Wallace, the naturalist and co-originator of the theory of natural selection who in his later years showed a passion for anti-vaccinationism and spiritualism. Personally troubled by Russell Wallace’s elementary statistical distortions and strained interpretations, McVail concluded that these provided clues to the anti-vaccinationists’ essentially distorted mode of reasoning.^115^ A number of his colleagues fell into the habit of attributing the problem to a mental or moral defect. ‘I have often wondered’, remarked Dr Robert Cory, ‘what motive they can possibly have. Why should they work against truth, reason and humanity?’^116^

Any answer to that line of questioning would hardly prove satisfactory, but this did not prevent several highly charged and sweeping characterisations. Having also tangled with Russell Wallace in more than a few newspaper battles, Bond suggested the eminent naturalist would provide an excellent subject for careful ‘psychological study’. It might clarify, for instance, how ‘his intellectual idiosyncrasies and emotional susceptibility serve only to mislead him into fallacies which men of much less originality can easily perceive and avoid’.^117^ Several ‘scientific men’ felt ‘ashamed’ to find Russell Wallace among the anti-vaccinationists; German zoologist Ernst Haeckel lamented that the celebrated naturalist’s mind must have ‘become diseased’.^118^ Broad generalizations about mental and emotional confusion became pervasive. *The British Medical Journal* felt it sufficient to condemn the membership of the Anti-Vaccination League as ‘a fraternity whose predominant quality is a preternatural dullness of apprehension’.^119^ They were branded as ‘pseudo-scientific’ meddlers, just as this term was shifting from its earlier meaning of erroneous science to the modern connotation of stubbornly misguided and innately biased inquiry.^120^ The professor of pathology at Harvard University contended that anti-vaccinationists as a group appeared as ‘a class of people who are either ignorant, or have a peculiar order of mind which renders them incapable of sane judgement’.^121^ This rhetorical style bore close similarity to wholesale dismissals of the female mind. One particularly unsubtle critic combined the two, declaring that the anti-vaccinationists ‘stuff themselves on literature compiled by ignorant, hysterical women’.^122^ The ‘queer doctrine’ fanned by these ‘dangerous fanatics’, in the opinion of another editorialist, could only be explained as an ‘incurable aberration of intellect’.^123^ Bond himself denounced the ‘few wealthy perverts’ who largely subsidized the National Anti-Vaccination League.^124^

There was no hope of converting these die-hards, Bond advised, but it was necessary to guard against others being misled by their ‘defective logic and incorrect assertions’.^125^ For reasonable people, epidemics like Gloucester’s had provided ‘an object lesson which nothing but the most firmly rooted prejudice could fail to comprehend;’ while, on the other hand, those who refused to understand proved that their ‘incredulity is hopeless’.^126^ Still, Bond cautioned against concluding that these dogmatic anti-vaccinationists were ‘ignorant fools’, as many were inclined to assume. In several writings, he dwelt on the ‘curious psychological phenomenon’ wherein otherwise intelligent and sane persons remain prisoners of a narrow and specific irrational prejudice.^127^ His many public clashes with Walter Hadwen had left Bond with one strong impression – that this was an example of a person who felt ‘no sense of moral responsibility’ for the unscrupulous repetition of false statements. It could be ‘only charitably explained by a perversion of intellect which is quite compatible with considerable acuteness in other respects’.^128^ In a published exchange with the President of the National Anti-Vaccination League, Bond suggested that his opponents suffered from ‘a form of mental aberration’ and likened it to a defect in vision such as colour blindness.^129^

It was increasingly common at the turn of the century to draw upon the new lexicon of mental disorders and neuroses to explain social and political problems, even though these still mostly rested on rather loose judgements of moral perversion and character defect. Psychologists employed the figure of the anti-vaccinationist for clinical descriptions of conditions such as monomania and the *idée fixe* and classes like paranoiacs and hypochondriacs.^130^ The experimental psychologist Joseph Jastrow, who aimed to construct a psychology of erroneous beliefs, considered anti-vaccinationists in the same light as those attracted to occultism and spiritualism. In his book, *The Psychology of Conviction*, they are described as captivated by sentiment against reason, a disposition that ‘paves the way for fanaticism’.^131^ Another English doctor classed anti-vaccinationists and similar enthusiasts as manifesting a sort of ‘neurotic diathesis’ shared by those predisposed to tubercular infection.^132^ Psychoanalytic interpretations of the anti-vaccinationist mind were less frequent, although the Assistant Physician to the Bethlehem Hospital did categorize them as ‘eccentrics’ suffering from a form of paranoia common in borderline cases of insanity. (He added, in the loose Freudian patter of the day, that it was related to ‘a psychosis erected on the invariable basis of repressed homosexuality.’)^133^

Derogatory caricatures became a reliable feature of the antipathy toward anti-vaccinationists. There was something abnormal in the ‘Physiology of the Anti-Vack’, an editorial in *The Practitioner* declared.^134^ This criticism resonated with growing anxieties over national and imperial decline, racial degeneration, moral decadence, and masculine enervation. Sir James Crichton-Browne, leading experimental psychiatrist and eugenics promoter, considered the anti-vaccinationist agitation ‘just another illustration of the spirit of lawlessness and defiance of authority’ afflicting England.^135^ He openly wondered whether persons who ‘by mental defect, or who by blind prejudice’, rejected vaccination might be ‘weeded out’ in the struggle for existence.^136^ Likewise, one public vaccinator suggested that the ‘the propagandists of the cult’ suffered from deranged or primitive neural development but that it should be evolutionarily corrected by their eventual higher mortality.^137^

This pathologization of the ‘typical’ anti-vaccinationist appeared alongside another immodest generalization – that these agitators were part of a conspiracy against progress and expertise, practically a war on science. The Jenner Society’s first annual report stated that the defence of vaccination was critical to upholding the work done by modern bacteriologists, who promised discoveries just as important as Jenner’s had been. They had been attacked with the same ‘fanaticism’ and by ‘precisely the same class of people’ against smallpox vaccination, it explained, adding that ‘the names and work of JENNER and PASTEUR must stand or fall together’.^138^ To be sure, there was a significant amount of overlap between English anti-vaccinationists and kindred movements such as anti-vivisectionism, spiritualism, feminism, and others; however, the most vocal anti-anti-vaccinationists exhibited an unsubtle zeal to frame these as nothing other than mawkish assaults on reason itself. No less a figure than Major (soon-to-be Sir) Ronald Ross supported this assessment, stating that there was a ‘very low level of intellect’ that took up the campaign against vaccination ‘and against every kind of science’. He emphasized this opposition was the same sentiment that opposed ‘all forms of knowledge and all forms of science’.^139^

These characterizations primarily functioned as superficial disparagements rather than attempts to gain insight into the crisis of smallpox vaccination. This was the opinion of the Medical Officer of Health for Leicester, Dr C. Killick Millard, who complained of how the campaign against anti-vaccinationists had been carried on in what he called a ‘prejudiced and intolerant spirit’.^140^ Millard was a somewhat unorthodox public figure, as might be expected for one employed by the Leicester municipality. In several addresses and publications, he claimed that doctors and health officials had been overly assertive in their claims for vaccination and underappreciative of its harms. Even more pointedly, Millard questioned whether its startling decline could be entirely or even mostly attributed to the organised agitation of anti-vaccination groups. Instead, the ‘deep-rooted and intense hostility’ found in many places more likely stemmed from the heavy-handed machinery of state compulsion, backed by the arrogant attitude of the medical profession.^141^ Although certainly not an anti-vaccinationist, Millard professed a certain amount of sympathy. He claimed to read their literature and admitted that he had learned from it about the people’s real objections. For principled as well as practical reasons, he urged that the anti-vaccinator always be treated with ‘courtesy, consideration, and patience’. The spirited debate was valuable, he urged his colleagues, ‘but let us do so, as far as possible, with strict impartiality, and not as a partisan’.^142^ These views, along with his belief that it was time to end universal mandatory smallpox vaccination, ensured that Millard remained a marginal voice, and this speaks to the extent to which a combative obduracy had come to influence discussions of the issue in medical circles. Following the publication of one of Millard’s addresses in *Public Health*, a letter by the Newcastle Medical Officer of Health denounced it as an ‘illegitimate monstrosity’ and regretted that the journal should become a ‘dumping ground for eccentric notions’.^143^

While Bond personally maintained a more complex attitude toward professional self-criticism, he nonetheless upheld a remarkably unnuanced stance on expertise and the clout that he believed properly accrued to professional standing and respectability. In one of his last contributions to the controversy, Bond spelt out what he considered the meaning and importance of ‘trustworthy authority’. All the most eminent members of the medical profession strongly endorsed vaccination. Anti-vaccination doctors like Dr Hadwen were not only rare; as a group, they included none who was ‘of more than secondary position even in the hierarchy of medicine’. Meanwhile, the ‘militant combatants’ filling newspaper correspondence with their ‘shrill clamour’ and addressing ‘packed gatherings’ on platforms were, ‘from an authoritative point of view, mere nobodies’. On the other side were the ‘giants of sanitary science’ like Lister, Pasteur, and Koch, the ‘leading men in every branch of science throughout the world’, and numerous commissions ‘in all civilised countries’. How was it possible, Bond asked, that with ‘such an overwhelming contrast of authority before him’ any reasonable person should doubt their verdict? If the question were anything but vaccination, there would be no hesitation.^144^ Authority was a function of status, which must also serve as a determinant of trust. Bond’s tone hints at his exasperation that this logic did not hit its mark with the public more widely. It also underscores that he could not fathom why it did not.

## Conclusion

Despite being at the forefront of the defence of vaccination in England for roughly a decade, the Jenner Society’s ultimate impact is difficult to pinpoint. Outcomes were certainly not what Bond had hoped. The introduction of conscientious objection in 1898 did not immediately lead to a rise in non-vaccinations as many had feared; certificates of successful vaccination actually rose modestly for a few years, and some observers were quick to credit the Jenner Society’s work.^145^ Still, these trends were slowly reversing. Bond pressed for a bill that would have required re-vaccination at age twelve, and when there appeared to be no political appetite for this, he indignantly blamed the ‘party necessities of ambitious politicians’ and the intimidating ‘clamour of irreconcilable faddists’ in the Anti-Vaccination League.^146^ The landslide Liberal election in 1906 brought an influx of anti-vaccinationist-allied MPs to Parliament and marked a swing in momentum. The 1907 Vaccination Act enabled a parent to obtain exemption by making a statutory declaration instead of having to go into court, in effect making it easier to avoid vaccination than to comply. Compulsory vaccination was in effect dead – not an outcome necessarily opposed by Bond, but one that made vaccination goals significantly more difficult, especially as it was clear that elected officials were desperate to avoid becoming a ‘party to a campaign of persuasion’ about vaccination.^147^ Some years later, an advisor to the Ministry of Health complained that a serious effort had never been made by the Government to explain smallpox vaccination ‘by sympathetic educational crusade and not in the cold-blooded manner of the income-tax form’.^148^ The 1907 Act in fact marked the beginning of an accelerated national decline in infant smallpox vaccination rates – from about 70% to 50% by 1912, and 45% during the 1920s.^149^

But Bond had long since exited the stage as a vaccination controversialist, apparently disillusioned at the medical profession’s ‘policy of indifference’ toward his virtual one-man crusade.^150^ In fact, very little was heard from the Jenner Society after 1906. The title of anti-anti-vaccination champion passed next to Dr. Arthur Drury, public vaccinator for Halifax, who for several years carried on Bond’s quest to refute anti-vaccinationists whenever they appeared in the press. In 1909 Drury began editing a newsletter for public vaccinators called *The Jennerian*, and during his many terms as president of the Association of Public Vaccinators of England and Wales, he attempted to give it a more militant stance. The National Anti-Vaccination League itself experienced a substantial fall-off in donations and activity following their legislative victory in 1907. Drury considered it evidence of their ‘suffering from the want of persecution’.^151^ As a matter of fact, absent an enforceable vaccine mandate, there was diminished overall interest in the controversy, which carried on mainly at the periphery of public discourse. (*The Jennerian* was something of a mirror image of the *Vaccination Inquirer*, and vice versa, with large portions of both journals devoted to what the other side was up to.) Anti-vaccination as an organised movement waned further as a result of the Great War, during which ‘conscientious objection’ was widely stigmatized and the vaccination of servicemen went ahead largely unimpeded. Following Bond’s death in 1911 at the age of 78, the Jenner Society’s papers and accounts were handed over to Drury, who spent many years unsuccessfully petitioning the British Medical Association to help reactivate the organization.^152^ The Jenner Society was finally subsumed in 1920 within the Research Defence Society, a group dedicated to countering the anti-vivisectionists.

It goes almost without saying that the Jenner Society drew its meaning and purpose from what members saw as the profusion of medical misinformation. Consequently, as anti-vaccinationism lost momentum, anti-anti-vaccinationism also lost its relevance. Perhaps less obvious is that the activities of neither ‘antis’ nor ‘anti-antis’ bore a decisive relationship to vaccine hesitancy or vaccine compliance. Indeed, the National Anti-Vaccination League and the Jenner Society both dwindled while a certain type of public indifference toward smallpox vaccination continued to grow. This suggests that the connection of poor vaccination rates to the organised anti-vaccination movement was never quite as straightforward as Bond and the leaders of the medical profession were inclined to assume. By the 1920s English health officials had very few specific persons or groups to blame and could no longer consider anti-vaccinationist agitation a very important factor. There was also the awkward matter of declining vaccination rates not being directly tied to increases in smallpox, as health officials had confidently predicted.^153^ The Gloucester epidemic of 1896, which did so much to galvanize the Jenner Society message, ended up looking like an anomaly. One might say that England got lucky. The variety of smallpox that prevailed between 1901–04 was extremely mild, and another sudden jump in smallpox infections in 1922 (including Gloucester) had the ‘most trivial’ ever case fatality rate.^154^ Quite plainly, smallpox continued to disappear even though universal, compulsory, preventive vaccination had been essentially abandoned as a policy. On the other hand, despite health officials considering it a dangerous gamble, they could still rely on the people flocking to vaccination whenever smallpox cropped up as a credible threat.

We should be careful to not dismiss the influence of anti-vaccinationism as a movement. Certainly, this ambitious anti-medical propaganda campaign nurtured an atmosphere of doubt and indifference that far outlasted the heyday of activism from the 1880s to the early 1900s. But we should equally be aware that vaccine mistrust was largely driven by factors not fully appreciated by either anti-vaccinationists or anti-anti-vaccinationist crusaders like Francis Bond. Public attitudes toward vaccination during this time were complex and were shaped mainly at the margins by organised groups. Assuredly, we should also recognize the extent to which the mere spectre of the Anti-Vaccination League at the turn of the last century stretched pro-vaccine messaging into an odd shape. The medical profession, which struggled over whether it should even be openly debated, was generally content to let the Jenner Society wage the battles that ordinary practitioners assiduously ducked. The Jenner Society, meanwhile, was consumed with identifying vaccination’s enemies and rebutting (or, as was also often the case, ridiculing) them. The unresolved problem was that their rebuttal and ridicule remained largely premised on an abstract idea of professional authority and expert consensus which was not as widely shared as Bond assumed. The short but lively span of the Jenner Society thus provides a cautionary note about the style and substance of anti-anti-vaccinationism, then and now. It suggests that those who wish to promote vaccines should be alert to the ways that their messages can be negatively coloured by condescension. This history contains a lesson about how campaigns dedicated to fact-checking and exposing misinformation might too easily divert energy away from the hard work of building mutually respectful relationships between health professionals and the public.

## Footnotes

1 ‘Discussion of Vaccination in Gloucester,’ *Gloucester Citizen*, 9 February 1884, 4.

2 Not to be mistaken for the Edward Jenner Vaccine Society, founded in 2010 as a professional advocacy organization for academic vaccinologists.

3 Francis T. Bond, ‘The Vaccination Act,’ *The Standard*, 20 August 1898, 2.

4 Francis T. Bond, ‘The Jenner Society,’ *The Times*, 12 August 1898, 2.

5
*Pall Mall Gazette*, 13 August 1898.

6 The term was not generally used at the time, but suggested by at least one author. See ‘Pages from a Private Diary,’ *Cornhill Magazine* (January 1898), 167-8.

7 Key works include Nadja Durbach , Bodily Matters: The Anti-Vaccination Movement in England, 1853-1907 (Duke University Press, 2005); Stanley Williamson , The Vaccination Controversy: The Rise, Reign and Fall of Compulsory Vaccination for Smallpox (Liverpool University Press, 2007); Deborah Brunton , The Politics of Vaccination: Practice and Policy in England, Wales, Ireland, and Scotland, 1800-1874 (University of Rochester Press, 2013).

8 ‘Ten Threats to Global Public Health’ (WHO Spotlight, 2019) https://www.who.int/news-room/spotlight/ten-threats-to-global-health-in-2019

9

Paul Offit , Deadly Choices: How the Anti-Vaccine Movement Threatens Us All (Basic Books, 2011).

10

Federico Germani and Nikola Biller-Andorno , ‘The Anti-Vaccination Infodemic on Social Media: A Behavioral Analysis,’ PLoS ONE 16.3 (2021), e0247642.33657152 10.1371/journal.pone.0247642PMC7928468

11

Peter Hotez , The Deadly Rise of Anti-Science: A Scientist’s Warning (Johns Hopkins University Press, 2023).

12

Maya Goldberg , Vaccine Hesitancy: Public Trust, Expertise, and the War on Science (University of Pittsburg Press, 2021).

13 John Gibbs, *Our Medical Liberties, or, The personal rights of the subject, as infringed by recent and proposed legislation* (Sotheran, Son, and Draper, 1854).

14 John Simon, ‘Letter to the President of the General Board of Health, by the Medical Officer of the Board,’ in *Papers Relating to the History and Practice of Vaccination* (HMSO, 1857), xliii.

15 Durbach, *Bodily Matters*, 13-36.

16 E. C. Seaton, *Report from the Select Committee on the Vaccination Act (1867)*, C.246 (House of Commons, Printed 23 May 1871), 320, 22.

17

J. Thorburn , Vaccination: A Condensed Summary of the Evidence in its Favour and of the Objections Urged Against It (London: Simpkin, Marshall and Co., 1870), 38.

18

William Aitken , The Science and Practice of Medicine, v. I, second edition (Charles Griffin and Co., 1863), 272–3.

19 John Simon, *Report from the Select Committee on the Vaccination Act*, 193.

20 Robert Turner, ‘A Plea for Compulsory Vaccination Reform in Scotland,’ *The Practitioner* (February 1870), 94.

21 Durbach, *Bodily Matters*, 53-5.

22 H. Meredith Richards, ‘The Diagnosis of Small-Pox,’ *Public Health* (January 1902), 226.

23 E.C. Seaton, *Report from the Select Committee on the Vaccination Act*, 319-20.

24 ‘Tracts,’ *Vaccination Inquirer* (August 1880), 74.

25
*BMJ*, 20 December 1902, 1911.

26
*The Saturday Review*, 16 October 1869, 508.

27
*Facts Concerning Vaccination for Heads of Families* (National Health Society, n.d.); *Fact versus Fiction: A Refutation of the National Health Society’s Tract, Entitled, ‘The Prevention of Small-Pox’* (London Society for the Abolition of Compulsory Vaccination, October 1881); *Vaccination Inquirer* (June 1885), 42-3.

28 Charles Hopwood, *Hansard’s House of Commons*, 12 May 1893, c. 846; Sir Balthazar Foster, *Hansard’s House of Commons*, 19 September 1893, c. 1581-2.

29

Ernest Hart , The Truth about Vaccination: An Examination and Refutation of the Assertions of the Anti-Vaccinators (Smith, Elder, 1880), 1–2.

30 From 1880 to 1896 the London Society for the Abolition of Compulsory Vaccination.

31 Henry D. Dudgeon, quoted in *Vaccination Inquirer* (January 1887).

32 ‘The Vaccination Question – Anti-Meeting in Gloucester,’ *Gloucester Citizen*, 1 February 1896. See also Gloucestershire Archives (hereafter GA)/N27.19GS *The Case Against Vaccination: An Address by Dr. Hadwen at Goddard’s Assembly Rooms, Gloucester on Saturday, January 25, 1896* (Gloucester and District Branch of the National Anti-Vaccination League, n.d.).

33 GA/D5435.6.12 George Newman , To Parents & Citizens of Gloucester (Gloucester Branch of the National Anti-Vaccination League, 1896).

34 Francis T. Bond, *The Story of the Gloucester Epidemic of Small-Pox. Showing how it arose, the facts which make it notable, and the lessons it teaches* (The Jenner Society, 1896), 19.

35
*A Review of Certain Present Aspects of Smallpox Prevention* (Ministry of Health, 1931), 49.

36 GA/C26.8 Jenner Society, *First Annual Report, for 1896*, 3.

37 GA/C26.9 Jenner Society, *Report of the Executive Committee for the Year 1896*, 1.

38 Albert E. Cope, ‘The Educational Factor in the Vaccination Problem,’ *Journal of the Royal Sanitary Institute*, v.20 n. 3 (October 1899), 547.

39 ‘Nature’s School at Gloucester,’ *The Spectator*, 29 August 1896, 9.

40 F.T. Bond, ‘The Vaccination Problem: A Contribution to Its Solution,’ *Lancet*, 18 December 1897, 1581. This was also issued as a Jenner Society pamphlet: GA/V2.15 *The Vaccination Problem: A Contribution to It Solution* (The Jenner Society, n.d.).

41 Kristin E. Kondrlik, ‘Conscientious Objection to Vaccination and the Failure to Solidify Professional Identity in Late Victorian Socio-Medical Journals,’ *Victorian Periodicals Review* 53.3 (2020): 338-71. See also Nadja Durbach, ‘Class, Gender, and the Conscientious Objector to Vaccination, 1898-1907,’ *Journal of British Studies* 41.1 (January 2002), 58-83.

42
*The Times*, 18 September 1897, 9.

43 GA/ C26.16 Jenner Society, *Memorandum in Explanation of the Memorial to the President of the Local Government Board on the Subject of Vaccination Legislation*, n.d. See also GA/C26.11 Jenner Society, *Report of the Executive Committee for the Year 1897*, 2-3.

44 F.T. Bond, ‘Report of the Royal Commission on Vaccination,’ *Journal of the Sanitary Institute*, v. 18 (January 1898), 441.

45 Major Griffith, ‘Discussion,’ *Journal of the Sanitary Institute*, v.18 (January 1898), 453; F. T. Bond, ‘The Vaccination Problem: A Contribution to Its Solution,’ *Lancet*, 18 December 1897, 1581.

46 GA/N13.102.8 *The Vaccination Act, 1898: Meetings of the Executive Committee of the Jenner Society* (Gloucester: The Jenner Society, 1903).

47 Bond, ‘Report of the Royal Commission,’ 448; also *Lancet*, 25 December 1897, 1648.

48 Albert E. Cope, ‘The Educational Factor in the Vaccination Problem,’ *Journal of the Royal Sanitary Institute*, v.20 (October 1899), 548, 550.

49 Francis T Bond, ‘Disinfection,’ *Transactions of the National Association for the Promotion of Social Science* (Longmans and Green, 1879), 457-67; GA/V2.25GS Leaflets on Various Appliances Designed by Br. Bond; Janet Hudson, ‘Dr Francis Bond and a Stonehouse ‘Pioneer Enterprise,” *Stonehouse History Group Journal* (September 2017), 18-21.

50 ‘The Jenner Society,’ *The Practitioner* (January 1899), 8. See also ‘A Vaccination Crusade,’ *The Hospital*, 12 December 1896, 173; ‘Antivaccination Propaganda: Its Bane and Its Antidote,’ *BMJ*, 5 July 1902, 51.

51 GA/C26.13 Jenner Society, *Report of the Executive Committee for the Year 1898*, 6.

52 GA/C25.7 Jenner Society, *Report of the Executive Committee for the Years 1899 and 1900*, 12. See also ‘The Jenner Society,’ *BMJ*, 8 April 1899, 864.

53
*Vaccination Inquirer* (July 1885), 59.

54 Quoted in *Vaccination Inquirer* (September 1902), 107. See also ‘Vaccination in a Bad Way,’ *Vaccination Inquirer* (December 1887), 159.

55
*Vaccination Inquirer* (August 1888), 82; *ibid.* (July 1903).

56 ‘Antivaccination Controversy,’ *BMJ*, 21 April 1906, 942. See also *Vaccination Inquirer* (May 1902), 41; *ibid.* (May 1903), 42.

57 ‘An Epistolary Claque,’ *BMJ*, 20 November 1897, 1524-25; GA/C26.11 Jenner Society, *Report of the Executive Committee for the Year 1897*, 2-4.

58 Part of a Jenner Society scrapbook is held at the Wellcome Collection: ‘Gloucester Smallpox Epidemic 1896’ (unpublished scrapbook [1896]).

59 GA/D5435.6.12 The Committee of the Gloucester and District Branch of the National Anti-Vaccination League, *Dr. Bond’s Confessions* (12 May 1896).

60 James R. Williams, ‘Dr. Bond and the Vaccination Question,’ *Pall Mall Gazette*, 31 August 1898, 5; ibid., *Hereford Times*, 10 June 1899, 16.

61 ‘Antivaccination at Sidmouth,’ *BMJ*, 6 May 1899, 1117; J. M. Earle, ‘The Royal Commission and the Vaccination Act,’ *Public Opinion*, 23 September 1898, 391.

62
*The Times*, 2 November 1897, 7.

63 ‘Francis T. Bond,’ *Boston Medical and Surgical Journal*, 4 January, 1912.

64 ‘The Vaccination Act,’ letter from W. R. Hadwen, *The Times*, 13 September 1898, 13.

65
*Vaccination Inquirer*, 1 April 1899, 1.

66 ‘The Jenner Society,’ *BMJ*, 8 April 1899, 864.

67 ‘The Doctor’s Burthen,’ *BMJ*, 13 May 1899, 1173-4.

68 ‘Vaccination Legislation and the Jenner Society,’ *Lancet*, 26 February 1898, 591.

69 ‘The Case for Vaccination,’ *Lancet*, 15 January 1898, 171.

70 Francis T. Bond, ‘The Vaccination Act,’ *Times*, 30 August 1898, 13.

71
*Vaccination: The Case for and Against:* The Daily News *Debate between Dr. Bond and Mr. Alfred Milnes* (National Anti-Vaccination League, 1902), 6.

72 T.W. Hime, ‘Discussion,’ *Journal of the Sanitary Institute*, v.18 (January 1898), 463.

73 ‘Anti-Vaccination Meeting in Bristol,’ *Western Daily Press*, 25 March 1909, 6.

74 This lecture tour formed the basis for a major article. Elizabeth Garrett Anderson, ‘The History and Effects of Vaccination,’ *Edinburgh Review* (April 1899), 335-68.

75
*Vaccination Inquirer* (March 1903), 241. See also *Vaccination Inquirer* (June 1908), 53.

76
*Vaccination Inquirer* (June 1887), 33.

77
*Vaccination Inquirer* (June 1887), 33.

78
*Vaccination Inquirer* (January 1903), 197.

79 ‘The Vaccination Act,’ letter from W.R. Hadwen, *The Times*, 27 August 1898, 10.

80

Joseph S. Meisel , Public Speech and the Culture of Public Life in the Age of Gladstone (Columbia University Press, 2001); Martin Hewitt , ‘Aspects of Platform Culture in Nineteenth-Century Britain,’ Nineteenth-Century Prose 29 (Spring 2002), 1–32.

81 Francis T. Bond, ‘The Case for Vaccination,’ *Lancet*, 15 January 1898, 185. See also ‘The Agitation Against Vaccination,’ *The Medical Press*, 12 April 1899, 384.

82 Francis T. Bond, ‘The Dissemination of Knowledge Concerning Vaccination,’ *Lancet*, 8 March 1902, 698.

83 Francis T. Bond, *Notes on the Vaccination Controversy, More Especially for the Use of Those Proposing to Engage in Public Debates, Lectures, or Correspondence on the Subject*, reprinted in *The Jennerian*, 20 April 1912, 21-3.

84 GA/C26.8 Jenner Society, *First Annual Report, for 1896*, 3-4.

85 GA/C26.13 Jenner Society, *Report of the Executive Committee for the Year 1898*, 6.

86
*Gloucester Epidemic of Small-Pox, 1895-96. Report of the Committee Appointed by the Board of Guardians to Organize and Carry out the General Vaccination of the City and District* (Jenner Society, 1896), 25-6.

87 GA/C25.7 Jenner Society, *Report of the Executive Committee for the Years 1899 and 1900*, 12.

88 H. Donald M. Spence and Francis T. Bond, ‘The Jenner Society,’ *Medical Times and Hospital Gazette*, 7 November 1903, 716.

89 Albert E. Cope, ‘The Case for Vaccination, Illustrated by a Series of Diagrams, presented at the Annual Meeting of the Association of Public Vaccinators,’ *The Jennerian*, 20 February 1909, 660-2.

90 ‘Jennerian Literature,’ *BMJ*, 7 January 1899, 57; ‘Jennerian Literature,’ *The Medical Press*, 18 January 1898, 80.

91 Letter from Ellen Howell, *The Lancet*, 18 January 1902, 191.

92 GA/N13.102.7 *Two Objects Lesson in the Administration of Vaccination* (The Jenner Society, 1898); GA/N13.102.10 *What Lord Herschell Has Said on the Subject of Vaccination* (The Jenner Society, 1897); GA/N13.102.6 *Sanitation and Small-Pox. Declaration by Medical Officers of Health* (The Jenner Society, n.d.).

93 GA/NX27.1 *Dr. Creighton Confuted: The Experience of Gloucester* (The Jenner Society, n.d.).

94 GA/N13.102.7 *Two Object Lessons in the Administration of Vaccination, reprinted from The British Medical Journal* (The Jenner Society, 1898); GA/NX27.8 *Small-pox and Vaccination, the Lessons of the Two Latest Epidemics of Small Pox* (The Jenner Society, n.d.).

95 GA/N13.102.3 *Fifteen Reasons Why We Should Believe the Efficacy and Safety of Vaccination as a Protection Against Smallpox, and why we should adopt it for ourselves and our children* (The Jenner Society, n.d. [1899?]).

96 Rev. Walter Lloyd, *Sanitation and Small-Pox* (Gloucester: The Jenner Society, n.d.), reprinted from *Westminster Review* (November 1898), 548-60.

97 GA/N13.102.5 Violet Martineau, *A Question of Conscience, reprinted from Mother’s Union Journal* (The Jenner Society, n.d.).

98 [John McVail], ‘The Feeling of the Public with Regard to Vaccination, ‘*BMJ*, 23 May 1896, 1290.

99 GA/N27.20 *A Plea for the Children, with an Exact Reproduction of Two Pages of an Old Register Kept in the Parish Chapel at Pudsey in Yorkshire, in 1787 and 1792: Shewing (1) How Prevalent Small-Pox was in the Last Century; and (2) The Special Ravages It Made Amongst Children* (The Jenner Society, n.d.).

100 GA/N13.102.11 Jenner Society, *Some Unimpeachable and Unanswerable Object Lessons in what Vaccination does to protect against Small-Pox in a series of Illustrations* (The Jenner Society, n.d.); ‘The Jenner Society,’ *BMJ*, 25 February 1905, 431.

101 Hebert W.G. Macleod, ‘The Dissemination of Knowledge Concerning Vaccination,’ *Lancet*, 1 March 1902, 618-19. See also Herbert W. G. Macleod, ‘Discussion,’ *Journal of the Sanitary Institute* v.23 (April 1902), 26.

102 ‘A Memorable Incident of the Gloucester Epidemic,’ *BMJ*, 11 September 1897, 673.

103

Theodore M. Porter , Trust in Numbers: The Pursuit of Objectivity in Science and Public Life (Princeton, NJ: Princeton University Press, 1995)10.1177/03063129902900400711623934; Michael Friendly and Howard Wainer , A History of Data Visualization and Graphic Communication (Harvard University Press, 2021).

104 Albert E. Cope, ‘The Case for Vaccination, Illustrated,’ *The Jennerian*, 20 February 1909, 660-2.

105 T.D. Acland, ‘Vaccination and Common Sense: An Address delivered to the Annual Meeting of the Subscribers to the Hospital Saturday Fund at the Mansion House on March 22^nd^, 1902,’ *BMJ*, 26 April 1902, 1009-17.

106 GA/N13.102.2 *The Effect of Vaccination in Infancy on the Mortality from Small-Pox in Childhood: A Critical Test* (The Jenner Society, n.d.), 11.

107 ‘Indirect Vaccination,’ *The Spectator*, 9 November 1901, 692.

108 Francis T. Bond, ‘The Jenner Society,’ *The Times*, 12 August 1898, 2.

109 ‘Chipping Sodbury Guardians,’ *Bristol Mercury and Daily Post*, 26 May 1899.

110 ‘Barton Regis Guardians,’ *Bristol Mercury and Daily Post*, 27 May 1899.

111 ‘Leicester Board of Guardians,’ *Leicester Chronicle*, 20 May 1899, 2; *Leicester Chronicle*, 27 May 1899, 8.

112
*Hansard’s House of Commons*, 2 June 1899, c.187-284.

113 ‘Vaccinationists as Law-Breakers,’ *The Councillor and Guardian*, 24 June 1899, 612.

114 Sir James Crichton-Browne, *The Jennerian*, 16 January 1909, 557.

115

John C. McVail , Vaccination Vindicated: Being an Answer to the Leading Anti-Vaccinators (Cassell & Co., 1887), 90 . See also T. P. Weber , ‘Alfred Russel Wallace and the Antivaccination Movement in Victorian England,’ Emerging Infectious Diseases 16 (2010), 664–8.20350381 10.3201/eid1604.090434PMC3321934

116 Quoted in *Vaccination Inquirer*, 1 September 1886, 85.

117
*The Times*, 13 September 1898, 13.

118 Robert Cory, ‘Vaccination,’ *Nature*, 22 March 1888, 483; Ernst Haeckel, *The Evolution of Man, v. I* (Appleton and Co., 1897), 99.

119
*BMJ*, 24 March 1906, 694.

120 Arthur Hopkirk, *Anti-Vacc Veracities: A Commentary* (The Author, [1899]), 8.

121 William T. Councilman, ‘Smallpox,’ in *Modern Medicine: Its Theory and Practice, vol. 2: Infectious Diseases*, edited by William Osler (Lea Brothers, 1907), 251.

122 [William J. Robinson], ‘And Again Vaccination,’ *Critic and Guide* (February 1907), 3.

123 ‘A Jenner Society,’ *Bristol Times and Mirror*, 9 April 1896, 5.

124 GA/C25.7 Jenner Society, *Report of the Executive Committee for the Years 1899 and 1900*, 12.

125 Letter from F. T. Bond, *Hereford Times*, 17 June 1899, 16.

126
*Gloucester Epidemic of Small-Pox, 1895-96* (Jenner Society, 1896), 22–3, 40.

127 F.T. Bond, ‘Small-Pox,’ National Review (March 1902), 141–2.

128 Quoted in *Gloucestershire Echo*, 14 April 1896, 3.

129
*Vaccination: The Case for and Against:* The Daily News *Debate between Dr. Bond and Mr. Alfred Milnes* (National Anti-Vaccination League, 1902), 3.

130 ‘Insane Reformers,’ *Journal of Nervous and Mental Disease* v.11 (July 1884), 524.

131

Joseph Jastrow , The Psychology of Conviction: Study of Beliefs and Attitudes (Houghton Mifflin, 1918), 256, 258.

132 William Butler, ‘The Value from a Public Health Standpoint of the Declining Phthisical Death-Rate,’ *Public Health* (February 1899), 335.

133

W.H.B. Stoddart , The New Psychiatry: Being the Morison Lectures delivered at the Royal College of Physicians of Edinburgh in March 1915 (Balliere, Tindall and Cox, 1915), 61–2.

134 ‘The Physiology of the Anti-Vack,’ *The Practitioner* (May 1896), 454.

135 ‘Annual Meeting and Dinner,’ *The Jennerian*, 15 February 1913, 12.

136
*The Jennerian*, 16 January 1909, 555.

137 J. Foster Palmer, ‘On Certain Psychological Analogies in Relation to Vaccination and National Defence,’ *Public Vaccination Service Notes*, 20 February 1915, 7–10.

138 GA/C26.8 Jenner Society, *First Annual Report, for 1896*, 3–4.

139
*The Jennerian*, 20 November 1909, 402.

140 C. Killick Millard, ‘The Role of the ‘Anti’: An Apology and an Appeal,’ *Public Health* (January 1903), 219. See also Logie Barrow, ‘Epidemic City Fathers: Contagious Councillors?’ in *Keeping the Lid On: Urban Eruptions and Social Control since the 19^th^ Century* (Cambridge Scholars Publishing, 2010), 11-29; Ian Dowbiggin , ‘‘A Prey on Normal People’: C. Killick Millard and the Euthanasia Movement in Great Britain, 1930–55,’ Journal of Contemporary History 36 (2002), 59–85.10.1177/00220094010360010318077891

141 C. Killick Millard, ‘The Leicester method of Dealing with Small-Pox,’ *Public Health* (July 1904), 623. See also C. Killick Millard , The Vaccination Question in the Light of Modern Experience (H. K. Lewis, 1914), 167–8PMC230053120767004.

142 Millard, ‘The Role of the ‘Anti,” 219.

143 Henry E. Armstrong, *Public Health* (August 1904), 729–30.

144 Letter published in the *Bristol Daily Mercury*, reprinted in *The Jennerian*, 17 April 1909, 838.

145 ‘The Jenner Society,’ *Sanitary Record and Journal*, 29 October 1903, 434.

146 Jenner Society, *Report of the Executive Committee for 1903-04*, 2–3.

147 Arthur Drury, ‘Vaccination: A Plea for the Correction of the Misrepresentation of Its Detractors,’ *Journal of the Royal Sanitary Institute* (February 1909), 90.

148 J.C.G. Ledingham, ‘Discussion on Vaccination Against Smallpox in the Light of Recent Experience,’ *Proceedings of the Royal Society of Medicine* (February 1929), 513.

149
*A Review of Certain Present Aspects of Smallpox Prevention* (Ministry of Health, 1931), 48.

150
*The Jennerian*, 20 January 1912, 6; *ibid*, 16 March 1912, 13.

151
*The Jennerian*, 16 January 1909, 556.

152 Wellcome Collection, SA/BMA/F.58: British Medical Association, Vaccination Committee Minutes, 1906-1948.

153 At least 130,000 persons died of smallpox in England Wales between 1851 and 1880; the next thirty years claimed a few more than 20,000, and the next only 407. Major Greenwood , ‘The End of ‘Compulsory’ Vaccination,’ BMJ, 3 July 1948, 22–3.10.1136/bmj.2.4565.22PMC209022418871648

154
*A Review of Certain Present Aspects of Smallpox Prevention* (Ministry of Health, 1931), 8-9.

